# Genomic Prediction and Indirect Selection for Grain Yield in US Pacific Northwest Winter Wheat Using Spectral Reflectance Indices from High-Throughput Phenotyping

**DOI:** 10.3390/ijms21010165

**Published:** 2019-12-25

**Authors:** Dennis N. Lozada, Jayfred V. Godoy, Brian P. Ward, Arron H. Carter

**Affiliations:** 1 Department of Crop and Soil Sciences, Washington State University, Pullman, WA 99164, USA; den.lozada@wsu.edu (D.N.L.); jayfred.godoy@wsu.edu (J.V.G.); 2 USDA-ARS Plant Science Research Unit, Raleigh, NC 27695, USA; bpward2@ncsu.edu

**Keywords:** genetic correlation, genetic gains, genomic prediction, grain yield, high-throughput phenotyping, indirect selection, spectral reflectance indices

## Abstract

Secondary traits from high-throughput phenotyping could be used to select for complex target traits to accelerate plant breeding and increase genetic gains. This study aimed to evaluate the potential of using spectral reflectance indices (SRI) for indirect selection of winter-wheat lines with high yield potential and to assess the effects of including secondary traits on the prediction accuracy for yield. A total of five SRIs were measured in a diversity panel, and F5 and doubled haploid wheat breeding populations planted between 2015 and 2018 in Lind and Pullman, WA. The winter-wheat panels were genotyped with 11,089 genotyping-by-sequencing derived markers. Spectral traits showed moderate to high phenotypic and genetic correlations, indicating their potential for indirect selection of lines with high yield potential. Inclusion of correlated spectral traits in genomic prediction models resulted in significant (*p* < 0.001) improvement in prediction accuracy for yield. Relatedness between training and test populations and heritability were among the principal factors affecting accuracy. Our results demonstrate the potential of using spectral indices as proxy measurements for selecting lines with increased yield potential and for improving prediction accuracy to increase genetic gains for complex traits in US Pacific Northwest winter wheat.

## 1. Introduction

Trait phenotyping is a major constraint in plant breeding due to the number of lines that need to be evaluated in multiple environments and trials. Traditional phenotyping strategies are usually based on visual scores, which are costly, labor- and time-consuming, subjective, and sometimes destructive [1]. The time requirements and subjectivity of phenotypic selection may limit genetic gains achieved, or the improvement of a phenotypic value within a population after applying a selection strategy and is the product of additive genetic variation, selection intensity, and selection accuracy, divided by the number of years per cycle [2]. High-throughput phenotyping (HTP) has gained popularity as a fast, quantitative method to perform indirect selection toward increasing genetic gains in plant-breeding programs [2]. Using HTP sensor-based platforms such as near-infrared spectroscopy and canopy spectral reflectance resulted in a better understanding of the genetic basis of complex traits in a noninvasive, labor-efficient, and large-scale manner [3,4].

Improvement of grain yield is a priority for many wheat breeding programs [5]; however, due to its complex nature, breeding and selecting for lines with high yield potential is difficult. Secondary traits that are generally more heritable and are correlated with grain yield such as spectral reflectance indices (SRI) from HTP have been utilized for indirect selection of wheat lines with high yield potential [6,7]. SRIs are based on the changes in the pattern of reflectance and absorption at certain wavelengths of light [8] and are represented as ratios of the sum and/or differences at these wavelengths [4]. SRIs utilizing only near-infrared (NIR) wavelengths include the Normalized Water Index (NWI), whereas those including wavelengths in both the visible and NIR regions of the electromagnetic spectrum include the Normalized Difference Vegetation Index (NDVI) and Simple Ratio (SR) [7]. The Normalized Difference Red Edge (NDRE) index utilizes wavelengths from the red-edge region of the electromagnetic spectrum [9,10]. These values can be used to evaluate correlated traits associated with crop yield and productivity such as biomass, vegetation and water status, greenness, and the degree of senescence of plant canopies [11,12,13,14].

In comparison to phenotyping, genotyping has gained significant advances in previous years, mainly due to the expanding use of DNA-based marker platforms. For example, single nucleotide polymorphisms (SNPs) have allowed for inexpensive and efficient genomics-based breeding, quantitative trait loci (QTL) analyses, genome-wide association mapping, and marker-assisted selection of different crops [15,16,17,18]. One selection strategy that has gained considerable attention is genomic prediction, which uses genome-wide marker data to predict the breeding values of individuals in a test population [19]. A selection model is built using a training population, with known phenotype and marker data; this model is then applied to estimate genomic breeding values of selection candidates [20]. Genomic prediction is seen to complement association mapping in understanding the genetic complexity of important yield, agronomic, and disease-related traits [21,22,23]. Furthermore, genetic gain per unit time can be increased through genomic predictions as a result of reduced phenotyping requirement and selection at any stage of the breeding program [1]. Combining the power of HTP and genotyping has been demonstrated to leverage plant breeding to maximize genetic gains in selection, particularly in wheat [24,25,26,27].

Genetic improvement for traits via indirect selection, using secondary traits such as SRI, should be measured in genetic populations closely representing the area where the improvement is being targeted [13]. The Pacific Northwest (PNW) region is one of the major producers of wheat in the US; thus, improvement of current wheat varieties through phenotypic and genomic-based selection strategies in this area is necessary. The objectives of this study were to (1) determine the potential of using SRI for indirect selection of grain yield in PNW winter wheat; (2) develop a genomic prediction model to predict grain yield through independent validations with separate training and testing sets; and (3) evaluate the effects of including SRI as fixed effects in the model on the prediction accuracy for yield. A diversity panel of PNW winter-wheat lines was evaluated for SRI and yield in multiyear and multilocation trials and was subsequently used to train a model to predict the performance of Washington State University winter-wheat-breeding lines through independent validations.

## 2. Results

### 2.1. Phenotypic Correlations and Principal Components Analyses

**Figure 1:**
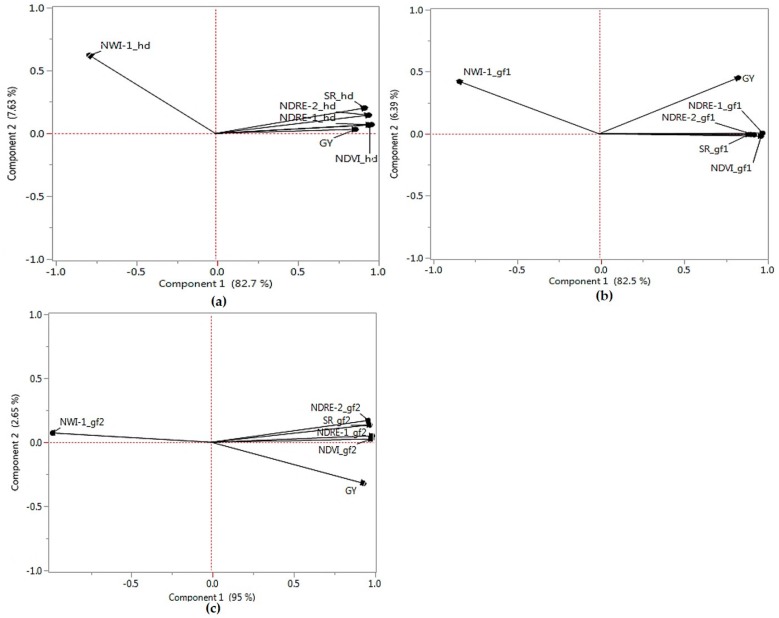
Principal component biplots for the spectral reflectance indices and yield across developmental stages for the Pacific Northwest winter wheat diversity panel. *Hd*- Heading (**a**); *Gf1*- Grain fill 1 (**b**); *Gf2-* Grain fill 2 (**c**). *GY*- Grain yield; *NDRE*- Normalized Difference Red Edge; *NDVI*- Normalized Difference Vegetative Index; *NWI*- Normalized Water Index; *SR*- Simple Ratio.

Significant (*p* < 0.001) phenotypic correlations of SRI with grain yield were observed across different developmental stages (Table 1) for the winter-wheat diversity panel (DP). In general, phenotypic correlation with yield increased from heading to the late grain fill (Gf2) across the indices. NDRE-1, NDRE-2, NDVI, and SR showed a positive significant correlation with grain yield and together formed a cluster in the principal components analysis (PCA) biplots (Figure 1). Phenotypic correlations among the visible and near-infrared indices ranged between 0.66 (SR and Gf1) and 0.86 (NDRE-1 and Gf2). NWI-1 had negative correlation with yield across developmental stages, ranging between −0.88 (Gf2) and −0.61 (Gf1). This negative correlation between the water-based index and grain yield was supported by PCA, where NWI-1 did not group with any other trait across the different growth stages. The first PC (PC1) explained between 82.5% and 95% of phenotypic variation, whereas PC2 explained between 2.7% and 7.6% of variation for the traits. Correlations for grain yield for the diversity panel and F5 and DH winter-wheat breeding lines were low, ranging between −0.11 and 0.26 (Table 2).

### 2.2. Heritability, Genetic Correlations, and Response to Selection

Broad-sense heritability for the spectral traits were low to moderate, ranging between 0.16 and 0.63 for NWI-1 (heading) and NDRE-1 (Gf1), respectively (Table 1). Heritability for grain yield was 0.19 (DP), 0.15 (F5_LND17), 0.13 (F5_PUL17), 0.56 (DH_LND18), and 0.53 (DH_PUL18). Except for NWI-1, *H*^2^ values were highest at the Gf1 stage. In the case of NDRE-1 and NDVI, higher *H*^2^ values were observed at Gf2 compared with heading. Mean spectral measurement values across locations decreased as the plants progressed to maturity, except for NWI. Conversely, phenotypic correlations between SRI indices and yield increased from heading to grain filling. Significant (*p* < 0.001) phenotypic and genetic correlations with yield were observed for all the measured SRI. Genetic correlations ranged between −0.58 (NWI-1 (Gf2)) and 0.67 (SR (heading)). Using NDVI, NDRE-1, and NDRE-2 resulted in a relative selection efficiency of greater than 1 (1.08, 1.17, and 1.07, respectively) for the first grain fill. Negative selection efficiency was observed for the water-based index NWI-1, ranging between −0.67 (Gf2) and −0.38 (heading).

### 2.3. Selection Based on Grain Yield and Spectral Reflectance Indices

The top 25% highest yielding lines (*n* = 115) were selected based on yield per se and SRI and the number of lines selected using both methods were compared. Selection based on SRI across environments resulted in correctly selecting 14% to 66% of the top 25% highest yielding lines across all environments (Table 3). Individually, 30% to 65% of the top 25% highest yielding lines were selected based on NDVI, whereas SR identified 28 to 65% of the top 25% highest yielding entries. The red-edge indices NDRE-1 and NDRE-2 selected 24% to 66%, whereas NWI-1 identified 14% to 66% of the top 25% highest yielding lines. Selecting for the top 25% highest yielding lines for the DP in 2017 and comparing their yield in 2018 resulted in a correlation of 0.08 (LND) and 0.14 (PUL).

### 2.4. Relationships between Actual and Predicted Grain Yield and Analysis of Principal Components

**Figure 2:**
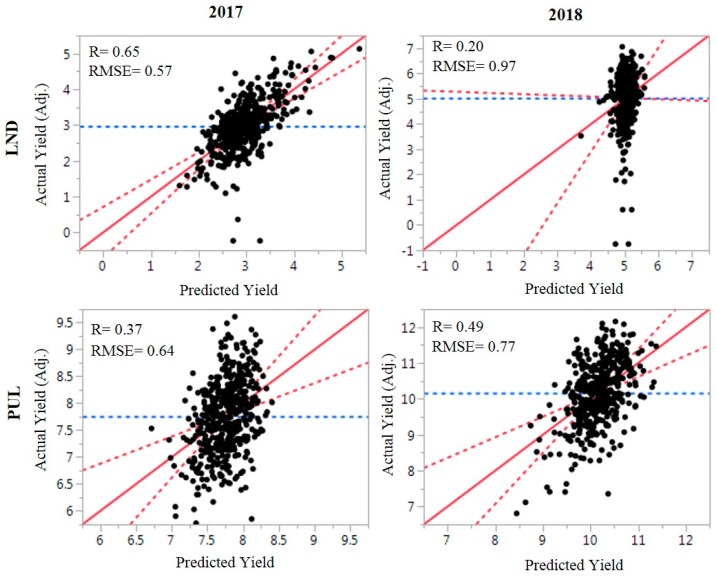
Relationship of adjusted and predicted yield based on least square regression models for LND and PUL, WA, 2017 and 2018 growing seasons. Spectral measurements across different growth stages were fitted to predict grain yield. Blue lines indicate mean grain yield, whereas solid and dashed red lines correspond of the line of fit for the regression model and the significant curve at *p* < 0.05, respectively. RMSE = root mean square error.

**Figure 3:**
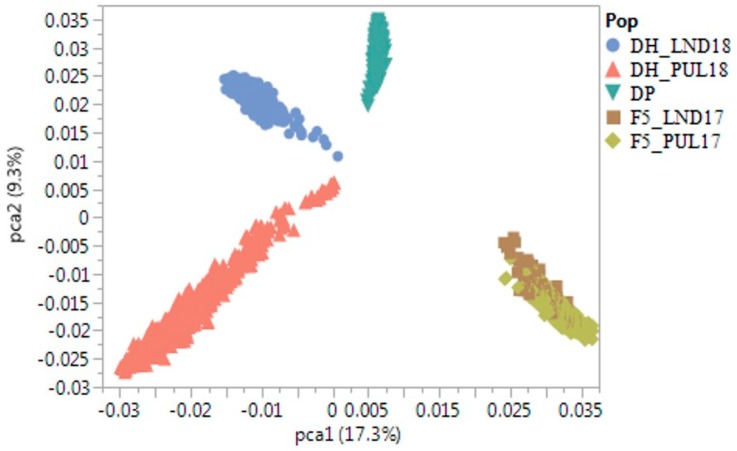
PCA biplot showing the genetic relationships of the winter wheat diversity panel (DP) with the DH and F5 wheat breeding lines used for genomic predictions with and without the presence of high-throughput secondary spectral reflectance traits as fixed effects in the model.

Correlation between actual and predicted yield derived from fitting the spectral measurements in LS regression models ranged between 0.20 (LND18) and 0.65 (LND17) (Figure 2). Root mean square error (RMSE) was lowest for LND17 (0.57), followed by PUL17 (0.64). Bayesian information criterion for the LS models for yield using spectral indices ranged between 843.98 (LND17) and 1327.31 (LND18). PCA biplots using SNP marker data for the winter wheat panels showed separation into distinct clusters based on population, where the diversity training panel did not group with the F5 and DH wheat breeding lines (Figure 3). The F5 lines grouped together on a single cluster. PC1 explained 17.3% of variation, whereas the second PC explained 9.3% of variation.

### 2.5. Prediction Accuracy for Grain Yield

**Figure 4:**
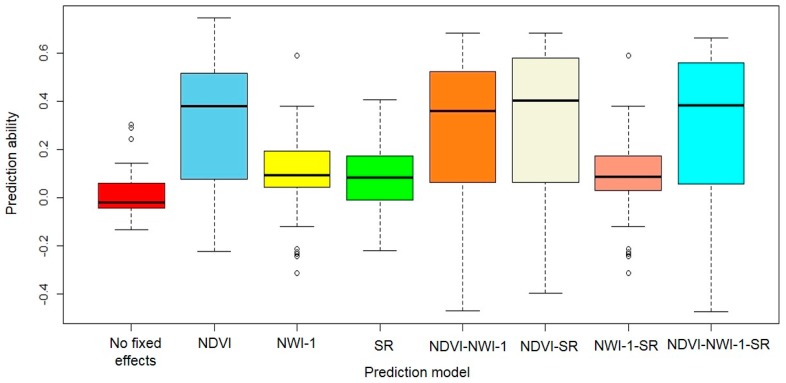
Boxplots for prediction accuracy for grain yield, using a diversity panel to predict the yield of DH and F5 winter-wheat breeding test lines in the absence and presence of secondary spectral traits as fixed effects in a ridge regression BLUP genomic prediction model. *NDVI*—normalized difference vegetative index; *NWI-1*—normalized water index-1; *SR*—simple ratio.

Significant differences (*p* < 0.001) were observed for mean genomic prediction accuracy with and without the spectral measurements as fixed effects in the model for both CV and independent predictions (Figure 4). Average values for prediction accuracy for yield under CV using the DP were 0.38 (without fixed effects) and 0.43 (with fixed effects). The number of traits included in the model did not significantly impact genomic prediction accuracy under CV. Inclusion of NDVI, NWI-1, and SR resulted in prediction accuracy ranging between 0.33 and 0.66. Using two spectral traits in the model led to average prediction accuracy of 0.43 across all trait combinations (NDVI + NWI-1, NDVI + SR, and NWI-1 + SR), whereas incorporating NDVI, NWI-1, and SR together resulted in a mean prediction accuracy of 0.42 for yield.

Mean prediction accuracy was 0.01 and 0.21 for models without and with the presence of SRI as fixed effects, respectively, for the independent predictions. Inclusion of NDVI, NWI-1, and SR alone in the prediction model led to mean prediction accuracies of 0.32, 0.09, and 0.08, respectively, whereas using all traits in the model resulted in a mean prediction accuracy of 0.29 for the independent predictions. Using two traits resulted in average prediction accuracy of 0.09 (NWI-1 + SR), 0.27 (NDVI + NWI-1), and 0.33 (NDVI + SR). Predicting yield for the DH populations resulted in higher prediction accuracy compared with using the F5 lines, with or without the fixed effects in the model.

Using one, and two and three SRI led to a 75% and 42% increase in prediction accuracy, respectively, for the DH lines relative to using the F5 as test populations. Roger’s distances between the diversity training panel and the DH lines was 0.29, whereas genetic distance between the DP and the F5 populations was 0.32. Using the DH populations as validation sets resulted in a higher mean prediction accuracy (0.23) compared with using the F5 panels (0.15) with and without the presence of spectral traits as fixed effects in the prediction model.

Using the LND18 dataset as training population showed the highest average prediction accuracy when fixed effects, either as a single trait or in combination with other indices, were included in the model (0.34), followed by using LND_Com (0.30) for the independent predictions. Overall, no significant difference was observed when using the combined datasets (LND_Com and PUL_Com) and using single environments as training sets for predictions (mean accuracies of 0.22 and 0.21, respectively) in the presence of fixed effects. Accuracy within similar environments was significantly higher (*p* < 0.05) compared with the accuracy for different locations (0.22 vs. 0.15). Prediction accuracies within LND environments were significantly higher (*p* < 0.0001) compared with the predictive accuracy within the PUL environments. Using LND to predict PUL and PUL to predict LND resulted in mean accuracy of 0.11 and 0.18, respectively.

## 3. Discussion

In this study, we have demonstrated the potential of using spectral reflectance traits from HTP to select for winter wheat lines with high yield potential in US PNW growing conditions. Different parameters, such as genetic and phenotypic correlations, responses to selection, correlated responses, and relative efficiency of selection for different spectral traits, were assessed in order to determine their efficiency for indirect selection for grain yield. In addition, the effect of including these traits as fixed effects on the accuracy of genomic prediction models was evaluated through cross validations within a diversity training panel and through independent predictions, where the diversity panel was used to predict grain yield in DH and F5 winter wheat breeding lines.

### 3.1. Indirect Selection for Grain Yield, Using Spectral Reflectance Indices

Secondary traits, such as the spectral indices from high-throughput field phenotyping, have been previously explored as substitute measurements for indirect selection of grain yield in wheat [13,14,28]. The success of indirect selection mainly depends on heritability and genetic correlation between the primary and secondary characters [29]; hence, correlated traits with higher heritability than the target trait have great potential for indirect selection.

Positive genetic and phenotypic correlations of NDRE, NDVI, and SR with grain yield were observed, consistent with reports in both bread and durum wheat evaluated under different environments with contrasting moisture regimes [11,30]. These positive associations were further supported by PCA plots, where these indices clustered with yield across developmental stages (Figure 1). In contrast, negative correlations with yield were observed for water index (NWI-1), similar with previous observations in wheat [8,12]. Higher values for water index demonstrate a lower water status in the canopy, suggesting an increased water stress at later growth stages [31] and an overall decrease of water content at the late grain fill. Phenotypic correlation values tended to increase as growth stage progressed from heading to the late grain fill stage, similar with reports in wheat [6,8] and in soybean [32]. Genetic correlations between SRI and yield were moderate to high, with positive correlations observed for NDVI, NDRE, and SR, and negative relationship with the water-based index NWI-1. SRI-yield correlations at heading and early grain fill stages were generally higher, indicating that the shared genetic components between SRI and yield were greater at these developmental phases [7]. The moderate to high genetic correlation of SRI with yield in this study demonstrates their potential for indirect selection. When selecting for yield, the importance of taking measurements at heading and grain filling (at a minimum of one measurement for each stage) to efficiently distinguish lines for yield selection was previously highlighted [31]. The observed genetic correlations of SRI with grain yield across different developmental stages in the current study further support the recommendation of Babar et al. [31] of taking measurements in the heading and grain fill stages for selection of high yield potential lines.

### 3.2. Heritability of the Spectral Reflectance Indices across Different Developmental Stages

Broad-sense heritability values were highest at the first grain fill for all traits, except for the water-based index NWI-1. The SRI in general had higher *H*^2^ than yield, further indicating the potential to be used for indirect selection, and that sustainable genetic gains could be attained by incorporating secondary traits during selection [12]. Low *H*^2^ values for some of the SRI measured in this study were expected, as it is typical for diverse populations in un-replicated trials to have low *H*^2^ values, similar with the results of Bowman et al. [8] for a diverse panel of spring wheat lines. Mean values for the SRI significantly decreased from heading to later maturity (grain filling) for NDRE-1, NDRE-2, NDVI, and SR, whereas a significant increase was observed for NWI-1 at later developmental stages. Spectral indices such as NDVI and SR indicate vegetative biomass and chlorophyll content, and hence depend on the quality of the plant tissues [6]. Senescence at the later growth stages could then cause a reduction in values for SRI as plants mature, leaves senesce, and photo-chemicals are recycled [6,8]. Furthermore, there is a reduced leaf-area index in the grain filling stage and consequently, there would be a decrease in the reflectance at near-infrared but increases in the reflectance of visible wavelengths. Hence, decreased values for NDVI and SR are expected at later growth stages [6]. In the present work, 24% and 61% decreases in the means for NDVI and SR, respectively, were observed from heading to the second grain fill stage. The water-based index, NWI-1, in contrast, showed a 25% increase from heading to the second grain filling stage.

### 3.3. Genetic and Phenotypic Correlations and Relative Selection Efficiency for Grain Yield

NDRE is a less frequently used index to assess the vegetative health of plant canopies and could be utilized as an alternative to NDVI [10]. Comparable genetic and phenotypic correlations with yield for the visible and near-infrared (NDVI and SR) and the red-edge indices (NDRE-1 and NDRE-2) across growth stages were observed, with the peak value generally at the early grain fill. Nevertheless, the mean relative selection efficiency across growth stages was higher for NDRE, reaching a maximum of 1.17 for NDRE-1 (at Gf1), indicating that the red-edge indices could be more effective for indirect selection of yield in PNW winter wheat. On average, a 5% increase in selection efficiency across the growth stages was observed for NDRE-1 and NDRE-2 relative to NDVI. The red edge indices are not affected by the decrease in leaf-area index as the plant approaches maturity, and this gives the NDRE an advantage over conventional vegetation indices such as NDVI [9]. In a previous study in winter wheat, NDRE predicted dry weight and nitrogen uptake better than NDVI [33]. A stronger degree of linear relationship with biomass, nitrogen uptake, and grain yield was likewise observed for red edge-based indices for rice grown in the southern US [9]. Water-based indices, on the other hand, predicted yield better for wheat grown in US Great Plains rainfed environments [13] and at irrigated, drought-stressed, and high temperature conditions in Northwest Mexico [14]. In this study, however, we observed that NDRE and NDVI generally had higher heritability, phenotypic and genetic correlations, and response to selection, suggesting that they are more efficient than the water-based indices for indirect selection of yield in US PNW growing conditions. The higher correlations during grain filling compared with the heading stage further suggest that these are the optimal stages for indirect selection of lines with high yield potential, consistent with previous observations in diverse spring wheat lines [8].

Taken together, the spectral traits measured in this study had moderate to high heritability, genetic and phenotypic correlations with yield, which make them suitable for indirect selection. SRI, nonetheless, could be implemented not just as a standalone indirect selection tool but also under an integrated approach [7] alongside other strategies such as genomic prediction. Incorporating spectral measurements from HTP with genomic predictions has been demonstrated to improve accuracy for yield and increased genetic gains in wheat breeding programs [24,25,26].

### 3.4. Genomic Prediction for Grain Yield

Previous studies have shown the potential of increasing prediction accuracy for yield through the inclusion of fixed effects in the model, either in the form of genomic information for major genes, QTL associated with the trait of interest, or phenotypic data for correlated secondary traits. In soft winter wheat, Mason et al. [34] recently demonstrated that including markers diagnostic for photoperiod and vernalization loci improved accuracy for yield, using an RRBLUP model. Similarly, through cross validations, it was shown that using information for correlated traits such as kernel rating index could improve accuracy for predicting *Fusarium* head blight resistance in wheat [35]. Through simulations, Bernardo [36] showed that major genes responsible for ≥10% of genetic variance should be included as fixed effects in the model to increase prediction accuracy. Likewise, integrating major QTL as fixed effects in a ridge regression model and predicting independent samples improved accuracy for baking quality traits in winter wheat [37]. Galiano-Carneiro et al. [38] previously observed improved accuracy for *Fusarium* head blight-related traits in winter triticale (*× Tritosecale*) through fitting significant markers with >5% genetic variance as fixed effect in a weighted ridge regression model. Maximum gains in accuracy were also observed when combinations of multiple height and phenology genes were included as fixed effects in a prediction model for yield in a historical US winter-wheat panel [39].

In the current study, the inclusion of spectral traits in the model either alone or in combination resulted in a 13% gain in prediction accuracy under the CV. Overall, CV within a single winter-wheat panel resulted in a significantly higher mean prediction accuracy for grain yield, with and without the presence of spectral traits in the model compared with the independent predictions. This result was consistent with previous studies where CV showed higher predictive power than across-population independent validations [40,41,42,43]. Such greater power for CV could be the result of using common environments to predict closely related lines, and hence could introduce bias in prediction accuracy [44]. Independent validations using different training and testing panels could potentially avoid this bias and represent a scenario where the performance of new, untested lines from different environment(s) is evaluated. In soft winter wheat, the prediction accuracies of CV were observed to be higher compared with the independent predictions for grain yield and agronomic traits, potentially a consequence of the genetic relatedness among the lines used for genomic selection [45,46]. We observed a significant increase in prediction accuracy by incorporating spectral reflectance traits in the model (average of 0.01 and 0.21, in the absence and presence of SRI as fixed effects, respectively) through independent validations. NDVI showed superior prediction accuracy compared with using either NWI-1 or SR due to its high genetic and phenotypic correlations with yield (Table 1; Figure 1).

Using the more related DH panels as test sets resulted in higher prediction accuracy compared with using the F5 in the presence of SRI as fixed effects (mean prediction accuracy of 0.23 vs. 0.15), indicating the relevance of relatedness between the training and validation panels. This is consistent with previous studies that show the importance of using genetically related populations for predictions [27,47,48]. Our results further indicate that when genetically unrelated populations are used for genomic predictions, the inclusion of secondary traits in the model could increase prediction accuracy for grain yield. PCA biplots based on SNP markers showed the formation of distinct groups based on population type, where the diversity panel did not cluster with the breeding lines indicating that the training and test populations used for predictions are genetically unrelated (Figure 3). In addition to genetic relatedness between the training and test populations, trait heritability also influenced predictive power in the present study, similar with previous reports [42,49,50]. Predicting yield using the F5_LND17 (*H*^2^ = 0.15) and F5_PUL17 (*H*^2^ = 0.13) as test panels resulted in 35% lower prediction accuracy compared with using the DH datasets (*H*^2^ of 0.53 and 0.56 for DH_PUL18 and DH_LND18, respectively) for predictions with and without the presence of spectral traits in the model under independent validations. Altogether, the low prediction accuracy for yield observed in this study, particularly under the independent validations, could be a consequence of the lack of apparent genetic relatedness among the populations used, low to moderate heritability of the target trait, as well as the differences in the environments used for predictions. Using the water-based index NWI-1, which is negatively correlated with grain yield, resulted in similar prediction accuracy compared with using a positively correlated trait such as SR. In some datasets, using this index resulted in higher accuracies than using NDVI and SR, either alone or in combination. This indicates that a negatively correlated trait could still be used for indirect selection given that it has a significant and strong correlation with the target trait. Other prediction models for yield could also be developed to further assess how the inclusion of correlated secondary traits could affect prediction accuracy across multiple environments, years, and populations evaluated in the US PNW growing conditions.

It should be noted that adding secondary traits as fixed effects in the model is not always advantageous for increasing prediction accuracy. In the case of Moore et al. [51], for example, using kernel color as fixed effects did not improve accuracy for pre-harvest sprouting (PHS) in hard winter wheat, indicating that the trait per se was not a reliable predictor of tolerance to PHS. In the current study, low or negative prediction accuracies were observed particularly when performing predictions across different environments (i.e., LND predicting PUL and vice versa) even when SRI was included in the model for the independent validations. Predicting grain yield within similar locations resulted in a 47% advantage over predicting across locations. QTL effects vary under across-environment predictions and these differences resulted in reduced prediction accuracy [52]. The low phenotypic correlations between grain yield among the datasets used for predictions could have also resulted in reduced prediction accuracy (Table 3), consistent with previous observations by Huang et al. [52] in wheat. Our results thus indicate the importance of predicting in similar environments to attain optimal prediction accuracy, as genotype-by-environment interactions could result in lower accuracies for yield [24,50].

We were also interested in looking at the effects of the number of spectral indices modeled on the ability to predict grain yield. Altogether, we observed an increased prediction accuracy when all three spectral traits were included as fixed effects in the model compared with using only one or two traits in the CV and independent prediction scenarios. In the current study, the use of multiple genetically intercorrelated traits has been shown to increase model prediction accuracy. Incorporating NDVI, NWI-1, and SR resulted in an 81% and 26% increase in the prediction accuracy compared with using only one or two traits as fixed effects, respectively; whereas using two traits showed a 44% gain over using only a single index for predictions when performing independent validations. In contrast, the number of traits included in the model did not affect prediction accuracy for yield under CV. Crain et al. [26] previously observed that using both NDVI and canopy temperature for predicting yield in CIMMYT elite lines under a multivariate model resulted in a 7% gain in accuracy in comparison to using only a single trait, indicating an additive effect of including spectral reflectance traits on accuracy for yield. Prediction accuracy for yield was also improved by 70% through the inclusion of NDVI and canopy temperature in multivariate pedigree models in CIMMYT wheat lines [25]. In the current study, there were some cases, however, when using all three traits resulted in lower prediction accuracy compared with two-trait models, as in when PUL (i.e., PUL16, PUL18, and PUL_Com) datasets were used to predict F5_LND17 for the independent validations. Previously, it was observed that multiple trait models did not always result in improved predictions for sudden death syndrome resistance traits in soybeans [53] and biomass traits in sorghum [54]. Including two or more traits in the prediction model could result in lower accuracies as additional traits could introduce collinearity [55]. In this context, it would then be relevant to limit the number of traits to be included in the prediction models for yield. We observed that using NDVI and SR resulted in the highest mean prediction accuracy across the CV and independent prediction scenarios (0.35) and hence using these traits in combination could improve prediction accuracy for grain yield in US PNW growing conditions.

## 4. Materials and Methods

### 4.1. Plant Material

Five different panels of soft winter-wheat lines adapted to PNW growing conditions of the US were used in this study, which included a diversity panel (DP; *n* = 456 lines) consisting of cultivars and breeding lines from regional breeding programs. The other four panels consisted of winter-wheat breeding lines from Washington State University. These panels consisted of two F_4:5_ (F5) and two double haploid (DH) trials, each consisting of multiple crosses and multiple lines within each cross, as is common in many breeding programs. The DP was grown in Lind (LND) and Pullman (PUL), WA in 2015–2018, whereas the F5 and DH panels were planted in 2017 and 2018 in LND and PUL, WA (F5_LND17, F5_PUL17, DH_LND18, and DH_PUL18).

The F5 and DH panels were grown as preliminary yield trials, with the purpose of selecting those lines with high yield potential to be advanced in the breeding program for additional replicated and multilocation testing. Spectral reflectance traits were measured in 2017 and 2018, whereas yield data was collected from 2015 to 2018 in LND and PUL, WA. The panels were planted in an augmented design [56] with repeated checks and un-replicated genotype in each block. ‘Eltan’ [57] and ‘Madsen’ [58] were used as checks in LND and only Madsen was used in PUL for the diversity panel for the 2015–2018 growing seasons. Eltan, ‘Xerpha’ [59], ‘Bruehl’ [60], ‘Otto’ [61], ‘Jasper’ [62], and Madsen were used as checks for F5_LND17; and Madsen, ‘Brundage’ [63], ‘UI Bruneau’, ‘Puma’ [64], Jasper, and Xerpha were used for F5_PUL17. Plots were 2.5 m in length, and 3.7 m^2^ in area, planted at a rate of ~260 plants per m^2^. Significant soil crusting impeding emergence was observed in LND in 2016 and hence the DP was not evaluated for SRI and yield on this site-year.

### 4.2. Collection and Analyses of Spectral Reflectance and Yield Data

Spectral reflectance data was collected using a CROPSCAN handheld multiple spectral radiometer (CROPSCAN Inc., Rochester, MN). The radiometer was attached to a pole and placed approximately 1 m above the canopy for taking measurements at the middle of each plot. Filters that reflect radiation at 16 different wavelengths between 430 and 970 nm were installed on the radiometer. Reflectance data were taken at three developmental stages: heading (Feekes stage 10.2), early grain-fill (milking stage; Feekes stage 10.7), and late grain-fill (soft dough stage; Feekes stages 10.9), with each measurement taken ~10–15 days apart. Spectral data collection was within a four-hour solar window (between 10:00 a.m. and 2:00 p.m.) on windless days with little or no cloud cover. Normalized Difference Red Edge-1 (NDRE-1), Normalized Difference Red Edge-2 (NDRE-2), Normalized Difference Vegetative Index (NDVI), Normalized Water Index (NWI-1), and Simple Ratio (SR) were calculated as follows: NDRE-1 = (*R*_800_ − *R*_700_)/(*R*_800_ + *R*_700_) [65]; NDRE-2 = (*R*_800_ − *R*_750_)/(*R*_800_ + *R*_750_); NDVI = (*R*_800_ − *R*_680_)/(*R*_800_ + *R*_680_) [66]; NWI-1 = (*R*_970_ − *R*_900_)/(*R*_970_ + *R*_900_) [12,13]; SR = *R*_900_/*R*_680_ [67], where *R*_n_ indicates reflectance at wavelength *n* of light (measured in nm). Grain yield (t·ha^−1^) was assessed by harvesting whole plots, using a Wintersteiger^®^ NurseryMaster combine.

Adjusted values were calculated for an augmented design, using the augmented complete block design with R (ACBD-R; [68]) in the R statistical computing environment [69] for individual environments and combined across environments. Best linear unbiased estimates (BLUEs) and predictors (BLUPs) were calculated for individual locations and for the combined analyses, respectively. Models used for calculation of BLUP and BLUE were as follows:

(1) $$Y_{ij}=\mu +B_{i}+G+C+I+\varepsilon_{ij}$$

and

(2) $$Y_{ijkl}=\mu +G+C+I+E_{i}+I\times E_{i}+G\times E_{i}+C\times E_{i}+B_{k}\left(E_{i}\right)+\varepsilon_{ijkl}$$

for individual location (1) and combined analyses across environments (2), where *Y* is the trait of interest; µ is the mean effect; *B*_i_ is the effect of the *i*th block; *G* corresponds to the un-replicated genotypes; *C* is the effect of the replicated checks on each block; *I* is the effect of check identifier; *E*_i_ is the effect of the *i*th environment; *E*_i_ × *C*, *E*_i_ × *G*, and *E*_i_ × *I* are the effects of environment by check, environment by genotype, and environment by check identifier interactions, respectively; *B*_k_(*E*_i_) is the effect of block *k* nested within environment *i*; and ε is a normally distributed residual effect with a mean of zero [68]. All effects were considered fixed when calculating BLUEs for individual locations, whereas effects were regarded as random when calculating BLUPs for combined analyses across environments.

Broad-sense heritability (*H*^2^) for SRI across all locations for each developmental stage and for grain yield across environments was calculated by considering genotype, replications, environment, and genotype by environment interactions as random effects, using the following formula: $H^{2}=\frac{\sigma_{G}^{2}}{\sigma_{G}^{2}+\frac{\sigma_{GEI}^{2}}{n}+\frac{\sigma_{\varepsilon }^{2}}{nr}}$, where $\sigma_{G}^{2}$ is the variance due to genotype; $\sigma_{GEI}^{2}$ is the variance due to genotype-by-environment interaction; and $\sigma_{\varepsilon }^{2}$ is the residual variance; *n* and *r* are the number of environments and replications per environment, respectively.

### 4.3. Correlation between Spectral Reflectance Traits and Grain Yield

Phenotypic correlations between SRI and grain yield were calculated by using JMP Pro v.11.0 [70]. Genetic correlation (*r_G_*), response to selection (*R*), correlated response (*CR*), and relative efficiency of indirect selection (*RE*) were calculated according to Falconer [71], using the following formula: *r_G_ =* $\left(Cov_{xy}\right)/\sqrt{\left(\sigma_{x}^{2}\times \sigma_{y}^{2}\right)}$, where Cov_xy_ is the covariance between traits *x* (SRI) and *y* (grain yield); $\sigma_{x}^{2}$ and $\sigma_{y}^{2}$ are the phenotypic variances of SRI and yield; *R* = *H*_x_σ_x_, where *H*_x_ is the square root of heritability for trait *x* (SRI), and σ_x_ is the genotypic standard deviation for trait *x* (SRI); *CR* = *H*_x_*r*_G_σ_y_; where *H*_x_ is the square root of heritability for trait *x* (SRI); r_G_ is the genetic correlation between SRI and yield, and σ_y_ is the genotypic standard deviation for yield; and *RE* = CR_x_/R_y_; where CR_x_ is the correlated response of the trait x (SRI) with yield; and R_y_ is the response to selection for yield. These parameters were calculated to estimate the efficiency of selection for grain yield using the spectral indices.

### 4.4. Predictive Models for Yield and Principal Components Analysis

To gain a better understanding of the relationships between secondary reflectance indices and grain yield in different developmental phases, predictions were conducted by fitting the spectral indices measured across growth stages as independent variables in least squares (LS) regression models for yield in JMP Pro v.11.0. Root mean square error (RMSE) was calculated for the yield models in four site-years (LND17, LND18, PUL17, and PUL18). The predictive LS model with the best fit was chosen by using the Bayesian Information Criterion. Relationships between adjusted and predicted yield based on the spectral measurements were visualized in biplots, using JMP Pro v.11.0. The top 25% of lines (*n* = 115) were selected based on yield per se and the SRI measurements in LND and PUL for 2017 and 2018 for the DP. The number of lines selected based on actual yield and SRI-based selection across environments were compared. PCA for SRI and yield across growth stages was conducted in JMP Pro v.11.0.

### 4.5. SNP Marker Genotyping and Genomic Predictions

The training and the test panels were genotyped, using genotyping by sequencing (GBS; [72]) through the NC State Genomics Sciences Laboratory in Raleigh, NC, using the restriction enzymes *MspI* and *PstI*. Sequences were aligned to the Chinese Spring International Wheat Genome Sequencing Consortium (IWGSC) RefSeq v1.0 [73], using the Burrows–Wheeler Aligner (BWA) 0.7.17 [74], followed by SNP marker calling, using TASSEL-GBS v. 5.2.43 [75,76]. After filtering for minor allele frequency (MAF > 0.05) and quality control, 11,089 GBS-derived SNP markers common to both training panel and test lines were used for genomic prediction. Out of this number, 10,894 SNP markers (98.2%) were with known chromosome (map) positions. Missing data imputation was done by using the ‘LDknni’ (linkage disequilibrium *k*-nearest neighbor joining imputation) function [77] implemented in TASSEL v. 5.2.25 software.

Genomic prediction accuracy for yield was assessed, using cross-validations (CV) using the DP and independent predictions. In the single population five-fold CV, 80% of the DP lines were used to predict the remaining 20%, with and without the presence of SRI in the prediction model. CV was conducted to assess the level of prediction accuracy which could be achieved prior to performing across-population predictions. Independent validations were then implemented, using the winter-wheat DP evaluated in LND and PUL, between 2015 and 2018, as training population and Washington State University F5 and DH winter-wheat breeding lines as test panels. The F5 panels evaluated in 2017 consisted of 61 (planted in LND; F5_LND17) and 501 (PUL; F5_PUL17) individuals, whereas the DH panels planted in 2018 comprised 449 (DH_LND18), and 761 lines (DH_PUL18). BLUE and BLUP estimates for yield and spectral traits (NDVI, NWI-1, and SR) were used for genomic predictions. Prediction accuracy was calculated as the Pearson correlation coefficient between the BLUE and BLUP estimates and the genomic estimated breeding values for grain yield. A total of nine datasets for the DP were used to train models to predict yield with or without the SRI as fixed effects in the model. Relatedness among the winter-wheat panels used for predictions was evaluated by calculating Roger’s genetic distance [78]. These genetic relationships were further visualized by using principal component biplots based on SNP marker data. All relatedness measures between the training and test lines were assessed by using JMP Genomics v.8.1.

A ridge regression best linear unbiased prediction (RRBLUP) model implemented in the ‘rrBLUP’ package [79] in R [69] through the iPAT software [80] was used for genomic predictions. This prediction model is represented in the form of y = **WGu** + ε, where **u** represents the vector of marker effects, **G** is a genotype matrix under an additive model, and **W** is design matrix relating lines to phenotypes or observations; ε is the vector of errors with variance σ^2^_ε_ [79]. Measurements for SRI as fixed effects were fitted in the model according to the equation y = X**β** + **Zu** + ε, where X is a design matrix for fixed effects **β**, and **Z** is the design matrix for random effects **u** [79]. The RRBLUP model follows an infinitesimal model and considers an equal variance for markers with effects reduced toward zero [1,81].

## 5. Conclusions

The present study showed the potential of indirect selection of high-yielding winter-wheat lines, using SRI collected from HTP. Moderate to high genetic and phenotypic correlation with yield, as well as generally higher heritability, were observed for the SRI, making them suitable for selection of lines with high yield potential. Altogether, relative selection efficiency at the first grain fill stages was higher, indicating that this is the optimal stage where indirect selection through SRI could be implemented. The red-edge indices, NDRE-1 and NDRE-2, could serve as alternatives to the more traditional vegetation indices such as NDVI, based on the relative efficiency of selection observed in the present study. Improved prediction accuracy under CV and independent validations were observed when SRIs were included as fixed effects to predict yield. Using indices in combination further provided better accuracies than using a single spectral trait for predicting yield. Integrating HTP and genomic prediction approaches could therefore facilitate increased genetic gains in winter-wheat breeding programs. Genomic predictions could help increase selection intensity and decrease the time to complete a breeding cycle, as selections would be based on the predicted genomic breeding values, consequently increasing gains achieved through selection. An increased selection accuracy resulting from incorporating HTP traits in the prediction model would also result in selecting lines with improved genetic performance. Overall, the relevance of using SRI as an indirect selection tool and their effects on increasing prediction accuracy was demonstrated, indicating that genetic gains for grain yield could be achieved for PNW winter wheat.

## Figures and Tables

**Table 1:** Heritability, mean, and genetic correlation with grain yield for spectral reflectance indices (SRI) across developmental stage measured for diverse Pacific Northwest winter-wheat lines.

Index ^1^	Growth Stage ^2^	Heritability	Mean	Correlation with Yield ^3^		Response to Selection ^4^	Correlated Response ^5^	Relative Selection Efficiency ^6^
				Phenotypic	Genetic			
NDRE-1	Hd	0.26	0.75	0.77	0.64	0.05	0.025	0.74
	Gf1	0.63	0.71	0.77	0.65	0.37	0.039	1.17
	Gf2	0.30	0.51	0.86	0.59	0.08	0.024	0.73
NDRE-2	Hd	0.42	0.14	0.78	0.65	0.10	0.032	0.96
	Gf1	0.53	0.13	0.73	0.65	0.12	0.036	1.07
	Gf2	0.30	0.10	0.81	0.58	0.08	0.024	0.72
NDVI	Hd	0.24	0.83	0.75	0.64	0.04	0.024	0.71
	Gf1	0.52	0.80	0.76	0.66	0.07	0.036	1.08
	Gf2	0.37	0.63	0.86	0.58	0.08	0.027	0.80
NWI-1	Hd	0.23	−0.08	−0.63	−0.51	0.04	−0.018	−0.56
	Gf1	0.16	−0.07	−0.61	−0.42	0.02	−0.013	−0.38
	Gf2	0.26	−0.06	−0.88	−0.58	0.06	−0.022	−0.67
SR	Hd	0.41	18.61	0.78	0.67	0.11	0.032	0.97
	Gf1	0.55	15.81	0.66	0.57	0.43	0.031	0.96
	Gf2	0.31	7.24	0.82	0.57	0.07	0.024	0.72

^1^*NDRE* = Normalized Difference Red Edge; *NDVI* = Normalized Difference Vegetative Index; *NWI* = Normalized Water Index; *SR* = Simple Ratio; ^2^ *Hd* = Heading; *Gf1* = Grain fill 1; *Gf2* = Grain fill 2; ^3^ all phenotypic and genetic correlations are significant at *p* < 0.001; ^4^ response to selection, R = H_x_σ_x_, where H_x_ is the square root of heritability for trait x (SRI); σ_x_ is the genotypic standard deviation for trait x (SRI); ^5^ correlated response, CR = H_x_r_g_σ_y_, where H_x_ is the square root of heritability for trait x (SRI); r_g_ is the genetic correlation with grain yield; σ_y_ is the genotypic standard deviation for yield; a higher CR would result in higher selection efficiency; ^6^ relative selection efficiency, RE = CR_x_/R_y_, where CR_x_ is the correlated response of the trait x (SRI) with yield; R_y_ is the response to selection for yield (equal to 0.033).

**Table 2:** Phenotypic correlations for grain yield for the diversity panel and the winter wheat breeding test lines.

Test Population	Diversity Panel								
	LND15	LND17	LND18	LND_Com	PUL15	PUL16	PUL17	PUL18	PUL_Com
F5_LND17	−0.08	0.26 *	−0.06	−0.03	0.11	0.19	0.24	0.22	0.19
DH_LND18	0.07	−0.03	−0.08	0.004	0.003	0.02	−0.02	−0.04	0.02
F5_PUL17	−0.06	−0.11	−0.01	−0.062	−0.03	0.02	−0.07	−0.02	−0.03
DH_PUL18	0.01	−0.01	0.04	0.03	0.04	0.09	0.06	0.07	0.05

* Significant at *p* < 0.05.

**Table 3:** Percentage of the top 25% (*n* = 115) highest yielding lines correctly selected using spectral reflectance indices across four site-years for a Pacific Northwest winter wheat diversity panel.

Index ^1^	LND17	LND18	PUL17	PUL18
NDRE-1	66.1	47.0	29.6	29.6
NDRE-2	66.1	46.1	26.1	31.3
NDVI	65.2	47.8	31.3	29.6
NWI-1	66.1	50.4	13.9	30.4
SR	65.2	45.2	31.3	27.8

^1^ NDRE—Normalized Difference Red Edge; NDVI—Normalized Difference Vegetative Index; NWI-1—Normalized Water Index; SR—Simple Ratio.

## Abbreviations

BLUE	Best linear unbiased estimate
BLUP	Best linear unbiased prediction
CV	Cross-validation
DH	Double haploid
DP	Diversity panel
HTP	High-throughput phenotyping
NDRE	Normalized difference red edge
NDVI	Normalized difference vegetative index
NWI-1	Normalized water index 1
NWI-2	Normalized water index 2
PCA	Principal components analysis
PNW	Pacific Northwest
QTL	Quantitative trait loci
RRBLUP	Ridge regression best linear unbiased prediction
SNP	Single nucleotide polymorphism
SR	Simple ratio
SRI	Spectral reflectance indices
